# An Assessment of Serological Assays for SARS-CoV-2 as Surrogates for Authentic Virus Neutralization

**DOI:** 10.1128/Spectrum.01059-21

**Published:** 2021-10-27

**Authors:** Nicholas Wohlgemuth, Kendall Whitt, Sean Cherry, Ericka Kirkpatrick Roubidoux, Chun-Yang Lin, Kim J. Allison, Ashleigh Gowen, Pamela Freiden, E. Kaitlynn Allen, St. Jude Investigative Team,, Aditya H. Gaur, Jeremie H. Estepp, Li Tang, Tomi Mori, Diego R. Hijano, Hana Hakim, Maureen A. McGargill, Florian Krammer, Michael A. Whitt, Joshua Wolf, Paul G. Thomas, Stacey Schultz-Cherry

**Affiliations:** a Department of Infectious Diseases, St. Jude Children’s Research Hospital, Memphis, Tennessee, USA; b Department of Microbiology, Immunology and Biochemistry, University of Tennessee Health Science Center, Memphis, Tennessee, USA; c Department of Immunology, St. Jude Children's Research Hospital, Memphis, Tennessee, USA; d Department of Hematology, St. Jude Children's Research Hospital, Memphis, Tennessee, USA; e Department of Global Pediatric Medicine, St. Jude Children’s Research Hospital, Memphis, Tennessee, USA; f Department of Biostatistics, St. Jude Children's Research Hospital, Memphis, Tennessee, USA; g Department of Microbiology, Icahn School of Medicine at Mount Sinai, New York, New York, USA; University of Georgia

**Keywords:** SARS-CoV-2, immunoassays, neutralizing antibodies

## Abstract

Severe acute respiratory syndrome coronavirus 2 (SARS-CoV-2) emerged in late 2019 and has since caused a global pandemic resulting in millions of cases and deaths. Diagnostic tools and serological assays are critical for controlling the outbreak, especially assays designed to quantitate neutralizing antibody levels, considered the best correlate of protection. As vaccines become increasingly available, it is important to identify reliable methods for measuring neutralizing antibody responses that correlate with authentic virus neutralization but can be performed outside biosafety level 3 (BSL3) laboratories. While many neutralizing assays using pseudotyped virus have been developed, there have been few studies comparing the different assays to each other as surrogates for authentic virus neutralization. Here, we characterized three enzyme-linked immunosorbent assays (ELISAs) and three pseudotyped vesicular stomatitis virus (VSV) neutralization assays and assessed their concordance with authentic virus neutralization. The most accurate assays for predicting authentic virus neutralization were luciferase- and secreted embryonic alkaline phosphatase (SEAP)-expressing pseudotyped virus neutralizations, followed by green fluorescent protein (GFP)-expressing pseudotyped virus neutralization, and then the ELISAs.

**IMPORTANCE** The ongoing COVID-19 pandemic is caused by infection with severe acute respiratory syndrome virus 2 (SARS-CoV-2). Prior infection or vaccination can be detected by the presence of antibodies in the blood. Antibodies in the blood are also considered to be protective against future infections from the same virus. The “gold standard” assay for detecting protective antibodies against SARS-CoV-2 is neutralization of authentic SARS-CoV-2 virus. However, this assay can only be performed under highly restrictive biocontainment conditions. We therefore characterized six antibody-detecting assays for their correlation with authentic virus neutralization. The significance of our research is in outlining the advantages and disadvantages of the different assays and identifying the optimal surrogate assay for authentic virus neutralization. This will allow for more accurate assessments of protective immunity against SARS-CoV-2 following infection and vaccination.

## INTRODUCTION

Severe acute respiratory syndrome coronavirus 2 (SARS-CoV-2) is the etiological agent of COVID-19 and responsible for a global pandemic and millions of deaths (1–4). Serological assays for detecting prior SARS-CoV-2 infection or vaccination are critical for containing the pandemic and assessing individual protection from future infection with SARS-CoV-2 (5–7). Authentic virus neutralization is considered the “gold standard” for detecting protective antibody responses, but for SARS-CoV-2, it can only be performed in biosafety level 3 (BSL3) laboratories (8–11). However, assessing protection from SARS-CoV-2, including variant strains, is critical for controlling the outbreak and determining when and how to lift COVID-19 precautions. While the majority of individuals who recover from SARS-CoV-2 infection have relatively long-lasting, protective immunity, a fraction do not mount protective immune responses and are susceptible to reinfection (12). It is therefore important to identify serological assays that can be performed outside BSL3 facilities yet still correlate with authentic virus neutralization and protection from reinfection.

Enzyme-linked immunosorbent assays (ELISAs) can detect antibodies against specific virus proteins or epitopes. Neutralization assays can be performed with authentic virus or a pseudotyped virus expressing the SARS-CoV-2 spike (S) protein on its surface and a marker to measure infection of cells (13, 14). The clear advantage of a pseudotyped virus is safety, as these studies can be performed in standard BSL2 laboratories. Another advantage is that results using pseudotyped virus can be obtained sooner (typically less than 24 h), whereas with authentic virus, plaque reduction-based neutralization assays take 2 to 3 days. A third advantage of using pseudotypes is flexibility. Pseudotypes expressing spike variants can be generated easily once the sequence is known since all that is needed is a plasmid that expresses the variant of interest. Additionally, pseudotyped viruses can be made to support multiple rounds of replication (15, 16), similar to authentic virus (17). One disadvantage of the pseudotyped virus neutralization assay is the pseudotyped viruses lack all but the spike protein from SARS-CoV-2, meaning they can only be neutralized by spike-specific antibodies, and the organization of proteins may not be representative of authentic virus particles. Yet, few studies have demonstrated whether the 50% neutralization dose (ND_50_), the dilution at which 50% of virus will be neutralized, differs between pseudotyped virus detection platforms and, importantly, how they compare to authentic virus (9, 10, 18, 19). Here, we characterized three enzyme-linked immunosorbent assays (ELISAs) and three pseudotyped VSV virus neutralization assays and assessed their concordance with authentic virus neutralization. The assays most predictive of authentic virus neutralization were luciferase (Luci)- and secreted embryonic alkaline phosphatase (SEAP)-expressing pseudotyped virus neutralizations. The next most predictive assay was green fluorescent protein (GFP)-expressing pseudotyped virus neutralization, followed by the ELISAs.

## RESULTS

### Study participants’ characteristics.

To fill this gap in knowledge, we compared SARS-CoV-2 antibody titers in 39 plasma samples from 34 individuals: 10 samples were taken from PCR-negative individuals, and 29 samples were taken an average of 36 days (interquartile range, 26 to 47) following a positive PCR test. Plasma was tested for antibodies against SARS-CoV-2 by ELISA, pseudotyped virus neutralization assay, and authentic virus neutralization. Adult participants were enrolled in the prospective, adaptive cohort study of St. Jude Children’s Research Hospital employees, “St. Jude Tracking of Viral and Host Factors Associated with COVID-19” (SJTRC) (clinicaltrials.gov no. NCT04362995), beginning in April of 2020. SJTRC was approved by the St. Jude Internal Review Board, and all participants provided written informed consent in a manner consistent with institutional policies. Cohort characteristics are provided in Table 1. Samples were collected between April and August 2020.

**TABLE 1:** Participant characteristics

Characteristic^*a*^	SARS-CoV-2 infection status	
	No infection (*n* = 10)	Infection (*n* = 29)
Age in yr, median (IQR)	48.5 (32.5–57)	45.5 (38–57)
Gender, *n* (%)		
Female	6 (60)	20 (83.3)
Male	4 (40)	4 (16.7)
Race, *n* (%)		
White/Caucasian	9 (90)	16 (66.7)
Black/African-American	1 (10)	7 (29.2)
Other	0 (0)	1 (4.2)
Ethnicity, *n* (%)		
Hispanic	0 (0)	2 (8.3)
Non-Hispanic	10 (100)	22 (91.7)

a All participant characteristics were self-reported.

### SARS-CoV-2 protein ELISAs.

The ELISAs included in the comparison detect antibodies to SARS-CoV-2 spike protein, nucleocapsid protein (N), or the receptor-binding domain (RBD) of the spike protein as described previously (20). Briefly, plasma samples were diluted 1:50 for RBD and N ELISAs, and results were expressed as the ratio of the optical density (OD) from the sample over that of the negative control (a known negative, prepandemic plasma sample), which is common practice. To determine spike titers, plasma was diluted 1:100 to 1:8,100 and an area under the curve (AUC) analysis performed. All PCR-positive participants had ELISA titers to RBD, N, and spike, although the titers differed (Table 2). The average RBD ratio for the positive participants was 16.96 (95% confidence interval [CI], 15.30 to 18.62), and it was 1.62 (95% CI, 1.33 to 1.91) for negative participants, while the average N ratios were 9.50 (95% CI, 8.03 to 10.96) for positive participants and 1.40 (95% CI, 0.79 to 2.01) for negative participants. The spike AUC average was 6.57 (95% CI, 5.40 to 7.75) for the positive samples (the spike ELISA was not performed on negative samples).

**TABLE 2:** SARS-CoV-2 protein ELISA values

Subject with PCR result	ELISA result for:		
	RBD ratio (sample/negative)	N ratio (sample/negative)	Spike value (AUC × 100)
Positive			
1	24.08	14.14	7.24
2	22.78	14.65	6.31
3	21.74	9.42	5.30
4	21.43	5.11	12.55
5	21.20	1.76	5.04
6	20.48	17.47	6.39
7	20.13	15.73	3.97
8	20.01	7.05	8.43
9	19.59	13.09	5.39
10	19.54	10.56	8.79
11	19.10	16.61	11.38
12	18.76	8.33	8.03
13	18.41	8.74	14.19
14	18.39	9.25	7.29
15	17.92	13.75	9.26
16	17.90	5.82	6.74
17	17.59	8.11	5.97
18	17.30	9.66	6.52
19	17.04	9.90	6.26
20	16.73	10.95	3.08
21	16.48	14.00	12.90
22	15.54	5.85	2.87
23	13.76	10.37	3.89
24	12.01	6.20	3.43
25	10.56	6.46	8.21
26	9.62	7.20	3.36
27	8.79	4.45	1.97
28	8.12	5.32	2.43
29	6.94	5.59	3.51
Negative			
30	2.27	3.57	
31	2.15	1.14	
32	1.99	1.01	
33	1.80	1.17	
34	1.75	1.25	
35	1.72	2.81	
36	1.45	0.68	
37	1.07	0.75	
38	1.04	0.71	
39	0.99	0.94	

### Authentic virus and pseudotyped virus neutralization.

**FIG 1:**
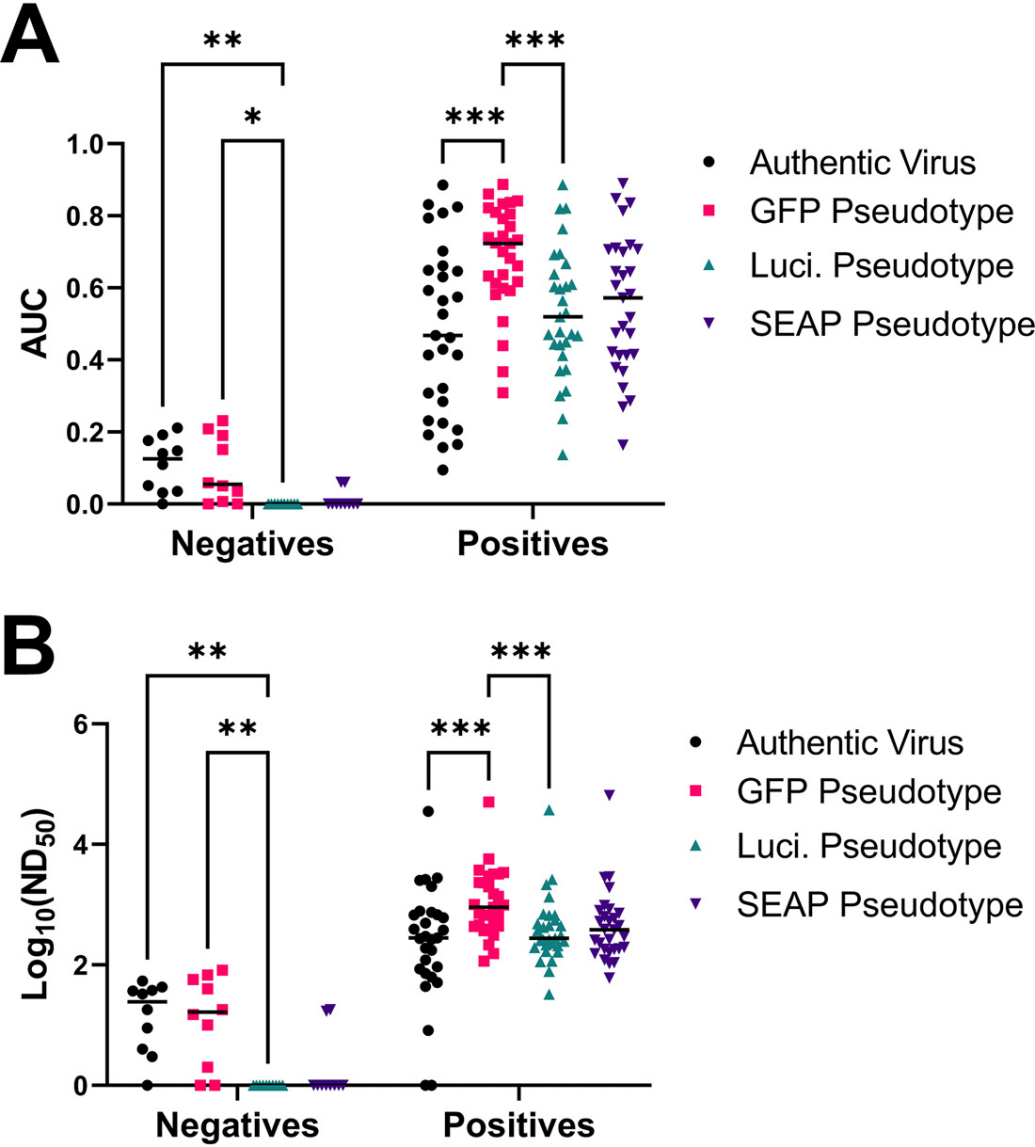
Comparison of neutralization assays by sample groups. Shown are area under the curve (AUC) (A) and 50% neutralization dilution (ND_50_) (B) calculations by neutralization assay type. AUC and ND_50_ values were calculated and used to compare authentic virus neutralization (black), GFP pseudotype neutralization (pink), luciferase pseudotype (teal), and SEAP pseudotype (purple). *, *P* < 0.05; **, *P* < 0.01; ***, *P* < 0.001 (mixed-effects model with the Geisser-Greenhouse correction and Tukey multiple-comparison posttest and *P* value adjustment). *n* = 34 samples run on each assay.

**FIG 2:**
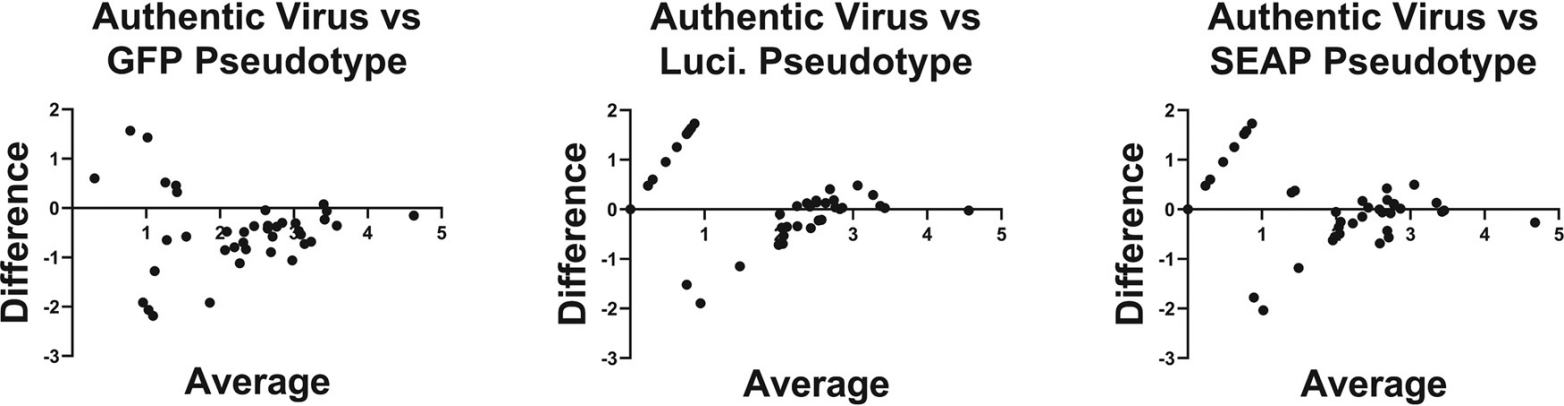
Bland-Altman analysis of SARS-CoV-2 pseudotyped virus neutralization assays. Bland-Altman analysis was performed between the log ND_50_ values of authentic virus neutralization against each pseudotyped virus platform. The difference between the two assays for each sample is on the *y* axis, and the average of the two assays is on the *x* axis.

The pseudotyped virus platform was a vesicular stomatitis virus (VSV) glycoprotein (G) knockout VSV expressing full-length SARS-CoV-2 spike protein (VSV-ΔG-S) from the Wuhan-Hu-1 strain with three different reporter genes: green fluorescence protein (GFP), luciferase (Luci), and secreted alkaline phosphatase (SEAP). Authentic virus neutralization studies were performed under BSL3+ conditions with the 2019n-CoV/USA_WA1/2020 strain obtained from BEI Resources. To quantitate neutralization titers, plasma was diluted from 1:100 to 1:900 and tested by authentic virus and VSV-ΔG-S GFP, Luci, and SEAP pseudotyped viruses. AUC and ND_50_ were calculated (Table 3). The average AUC values for the authentic virus neutralization assay were 0.485 (95% CI, 0.391 to 0.579) for positive participants and 10.96 (95% CI, 6.29 to 15.63) for negative participants. The GFP, Luci, and SEAP pseudotyped virus neutralization assays gave average AUC values of 0.685 (95% CI, 0.632 to 0.738), 0.530 (95% CI, 0.465 to 0.595), and 0.553 (95% CI, 0.483 to 0.622), respectively, for positive participants and 9.33 (95% CI, 3.62 to 15.04), 0 (95% CI, 0 to 0), and 1.21 (95% CI, 0 to 2.79), respectively, for negative participants (Table 3). The geometric average ND_50_ value for the authentic virus neutralization assay was 228.2 (95% CI, 98.66 to 527.8) for positive participants, and it was 13.56 (95% CI, 5.08 to 36.14) for negative participants compared to values of 1,052 (95% CI, 651.6 to 1,697), 375.3 (95% CI, 231.1 to 609.6), and 438.6 (95% CI, 261.3 to 736.2) for positive participants and 12.12 (95% CI, 3.562 to 41.27), 1 (95% CI, 1 to 1), and 1.772 (95% CI, 0.7476 to 4.202) for negative participants, respectively, for the GFP, Luci, and SEAP pseudotyped viruses. All neutralization platforms differentiated average negative and positive samples (Fig. 1). While the AUC and ND_50_ values were significantly higher for the GFP pseudotyped virus compared to the authentic virus or Luci pseudotyped virus, suggesting that VSV-ΔG-S-GFP could be a more sensitive assay, it is balanced by increased AUC and ND_50_ values in negative participants. Only the Luci and SEAP pseudotyped viruses showed no background in samples from PCR-negative participants. A Bland-Altman method comparison test shows that there is systematic bias between the different pseudotyped virus neutralization assays and the authentic virus neutralization assay, leading to higher variability (highest for GFP pseudotypes) when the signal is low for each assay (Fig. 2). However, this bias decreases when signal becomes higher, resulting in the pseudotype assays becoming more concordant with authentic virus neutralization. Finally, the average differences between the log ND_50_ for authentic virus neutralization and each pseudotyped virus neutralization are −0.481 for the GFP pseudotype, 0.129 for the Luci pseudotype, and 0.015 for the SEAP pseudotype.

**TABLE 3:** Authentic virus and pseudotyped virus neutralization summary statistics

Subject with PCR result	Authentic virus		GFP pseudotype		Luciferase pseudotype		SEAP pseudotype	
	AUC	ND_50_	AUC	ND_50_	AUC	ND_50_	AUC	ND_50_
Positive								
1	0.702	682	0.792	1,995	0.597	447	0.645	527
2	0.574	391	0.581	432	0.471	261	0.572	448
3	0.649	782	0.822	3,747	0.531	307	0.518	296
4	0.886	35,373	0.887	50,378	0.886	37,272	0.890	64,851
5	0.808	2,008	0.842	3,409	0.694	662	0.700	635
6	0.414	175	0.744	1,373	0.603	417	0.707	850
7	0.646	606	0.837	3,229	0.638	551	0.606	392
8	0.527	296	0.682	670	0.604	481	0.710	803
9	0.468	275	0.701	716	0.444	208	0.413	186
10	0.308	121	0.592	369	0.478	264	0.473	231
11	0.794	2,612	0.810	2,997	0.764	1,341	0.814	1,922
12	0.593	495	0.733	983	0.565	369	0.634	589
13	0.831	2,527	0.860	5,744	0.821	2,162	0.836	2,834
14	0.462	266	0.724	1,001	0.610	444	0.719	972
15	0.565	380	0.739	908	0.520	276	0.582	382
16	0.414	190	0.617	441	0.370	163	0.474	266
17	0.157	44	0.506	312	0.442	227	0.415	184
18	0.225	72	0.440	215	0.374	168	0.379	163
19	0.192	63	0.636	390	0.450	215	0.423	197
20	0.284	86	0.633	586	0.412	193	0.368	154
21	0.630	671	0.804	2,305	0.692	662	0.645	535
22	0.095	8	0.613	665	0.314	112	0.322	121
23	0.825	2,769	0.833	2,317	0.820	2,606	0.848	2,910
24	0.206	51	0.661	672	0.467	253	0.412	181
25	0.662	745	0.723	1,505	0.668	692	0.706	722
26	0.166	1	0.367	153	0.137	33	0.164	60
27	0.321	1	0.309	116	0.237	78	0.287	109
28	0.232	93	0.599	462	0.301	116	0.269	105
29	0.429	281	0.770	3,222	0.470	248	0.493	259

Negative								
30	0.176	43	0.051	15	0.000	1	0.060	18
31	0.035	3	0.191	57	0.000	1	0.000	1
32	0.192	37	0.000	1	0.000	1	0.060	17
33	0.141	33	0.035	10	0.000	1	0.000	1
34	0.051	4	0.000	1	0.000	1	0.000	1
35	0.2116	54	0.006855	2	0	1	0	1
36	0.1096	18	0.2085	68	0	1	0	1
37	0.1481	38	0.059	18	0	1	0	1
38	0.03123	9	0.1514	40	0	1	0	1
39	0	1	0.2311	82	0	1	0	1

### Comparison of serological assays.

**FIG 3:**
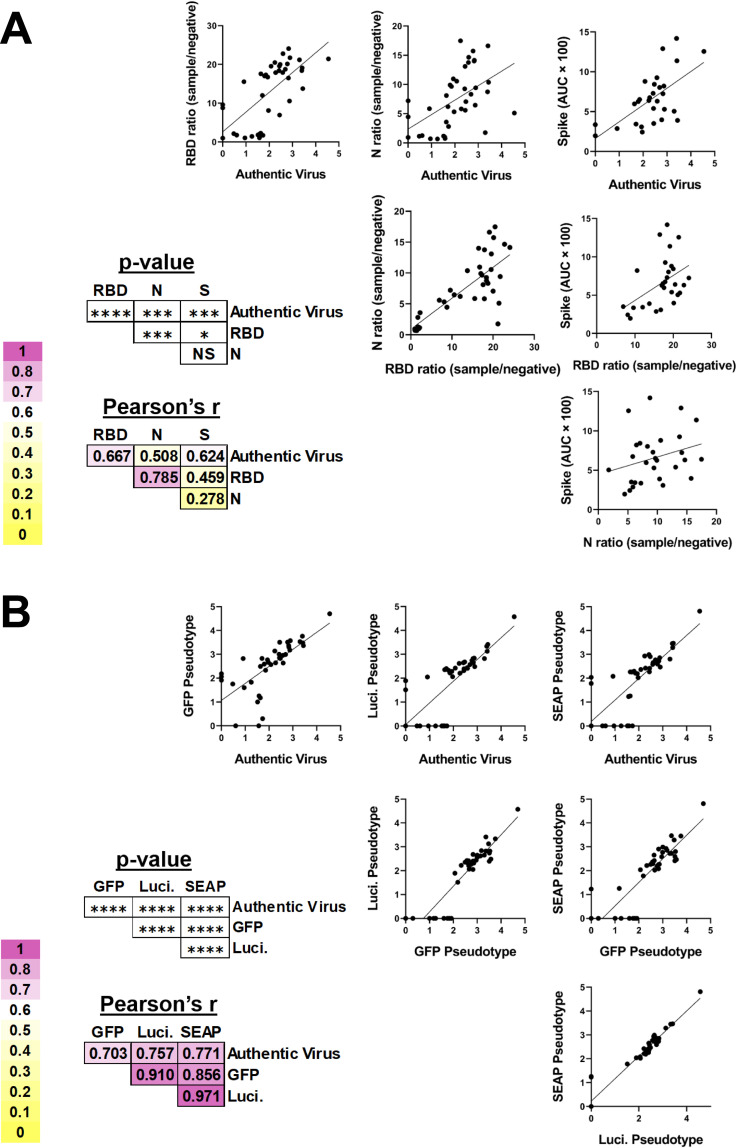
Correlation of SARS-CoV-2 serologic assays. (A) SARS-CoV-2-specific ELISAs and (B) VSV pseudotyped virus neutralization assays were compared by simple linear regression. The Pearson’s *r* values (a metric of correlation) and *P* values corresponding to each graph are to the lower left of each set of graphs. The background shading corresponds to the degree of correlation between the two assays. *, *P* < 0.05; ***, *P* < 0.001; ****, *P* < 0.0001.

**FIG 4:**
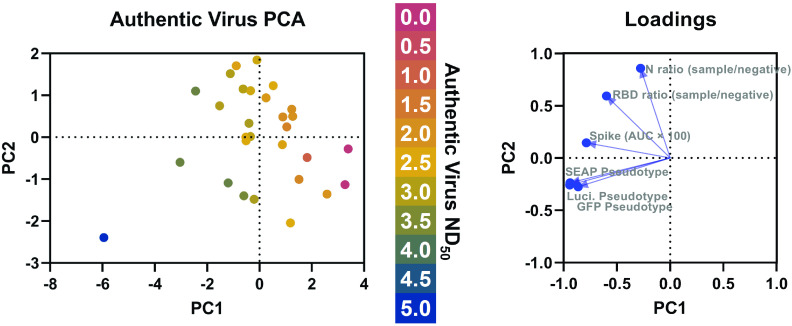
Principal-component analysis (PCA) of SARS-CoV-2 serological assays. Principal-component analysis was performed using all three ELISAs (spike, RBD, and nucleocapsid) and pseudotyped virus neutralization platforms (GFP, luciferase, and SEAP). The authentic virus ND_50_ is indicated by the color of the data point. PCA loadings generated during the analysis are shown on the right.

To determine which serological assays best correlated with authentic virus, linear regression analyses were performed, demonstrating that ELISA titers to the RBD (Pearson’s *r* = 0.667) and spike (Pearson’s *r* = 0.624) are significantly correlated with authentic virus neutralization titers (Fig. 3A). Nucleocapsid ELISA was significantly correlated with authentic virus neutralization, but had the worst correlation with authentic virus neutralization (Pearson’s *r* = 0.508), which has been shown previously for pseudotyped virus neutralization (21). This is also congruent with the observation that antibodies targeting the RBD domain of spike are highly neutralizing (22). Linear regression analyses demonstrated that all pseudotyped virus neutralization platforms were significantly correlated with authentic virus neutralization regardless of the reporter, with Luci (Pearson’s *r* = 0.757) and SEAP (Pearson’s *r* = 0.771) having the highest correlations (Fig. 3B). The pseudotyped virus neutralization assays were significantly correlated with each other, with Pearson’s *r* values as high as 0.971 between the Luci and SEAP assays. A principal-component analysis (PCA) was performed using all three ELISAs and all three pseudotyped virus platforms as variables (Fig. 4). The resulting PCA plot shows distinct clustering of the samples with the highest authentic virus neutralization titers and a gradient from poorly neutralizing samples (in the bottom left) to highly neutralizing samples (in the top right).

**FIG 5:**
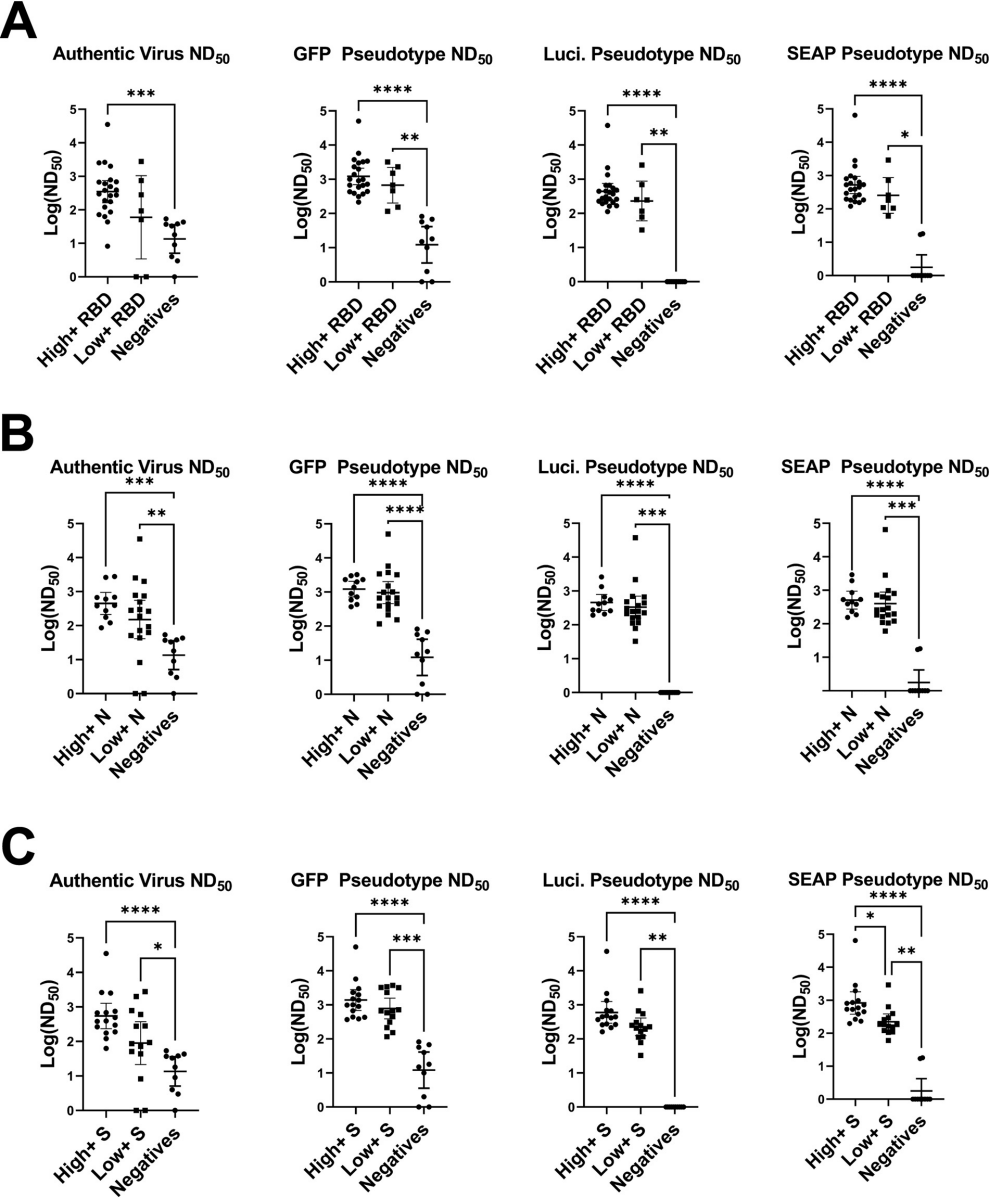
Comparison of high-positive, low-positive, and negative ELISA groups across neutralization assays. (A) RBD-, (B) spike (S)-, and (C) nucleocapsid (N)-positive samples were divided into high and low positives by finding cutoff values using histograms (RBD ratio, 15; N ratio, 10, and spike value, 6). Log ND_50_ values for the corresponding samples were then graphed and compared by Kruskal-Wallis test with Dunn's multiple-comparison tests. Significance thresholds: *, *P* < 0.05; **, *P* < 0.01; ***, *P* < 0.001; ****, *P* < 0.0001.

To assess granularity in the different ELISA results, cutoff values were used to categorize responses as high positive, low positive, or negative. Determination of cutoff values (RBD ratio of 15, nucleocapsid ratio of 10, and spike value of 6) was done by finding the internal nadir present in histograms for the different ELISAs. The stratification of RBD ELISA responses into high and low groups did not result in significantly different responses in any of the neutralization assays (Fig. 5A), suggesting that high RBD values do not necessarily correlate to higher neutralization titers, despite RBD ELISA positivity being associated with neutralization (Fig. 3A). Similar results were obtained for the spike ELISA (Fig. 5B) and nucleocapsid ELISA (Fig. 5C). There was, however, a trend for increased neutralization in the high-positive group versus the low-positive group for each neutralization assay, regardless of ELISA, justifying future studies specifically designed to test the granularity of these assays. Congruent with the findings in Fig. 3, highly positive ELISA results were significantly better at neutralizing than the negative samples for each ELISA.

## DISCUSSION

While all the serological assays were significantly correlated with authentic virus neutralization, some assays performed better than others at predicting authentic virus neutralization (Table 4). Based on correlation with authentic virus neutralization, the most accurate assays were the Luci and SEAP pseudotyped virus neutralization assays. GFP pseudotyped virus neutralization, the spike ELISA, and the RBD ELISA form a second tier of assays that are still quite accurate at predicting authentic virus neutralization. Furthermore, the GFP pseudotyped virus neutralization was able to detect antibodies at significantly higher dilutions than the other assays, making it the most sensitive assay tested. Despite nucleocapsid antigen being the basis for several common commercial antibody tests, nucleocapsid was the least predictive antigen of authentic virus neutralization.

**TABLE 4:** Logistical attributes of SARS-CoV-2 serological assays^*a*^

Assay	Accuracy	Technical requirements	Assay time	Price	Detection method
ELISA					
RBD	**++**	**+**	**+**	$	Enzymatic reaction
N	**+**	**+**	**+**	$	Enzymatic reaction
Spike	**++**	**+**	**+**	$	Enzymatic reaction

Neutralization					
Authentic virus	**+++**	**+++**	**+++**	$$$	Infectious unit
GFP pseudotype	**++**	**++**	**++**	$	Infectious unit
Luci pseudotype	**+++**	**++**	**++**	$$	Enzymatic reaction
SEAP pseudotype	**+++**	**++**	**++**	$	Enzymatic reaction

a The number of symbols (+ or $) is a relative estimate of the column variable. “Price” indicates the price of running the assay given access to all the technical requirements for the assay.

While the GFP virus was the most sensitive neutralization assay tested, it had higher background than the SEAP and luminescence assays. The sigmoidal relationship between the amount of analyte detected and the readout in SEAP and luminescence assays could be a reason for this difference. Variance at the lower end of the curve is less likely to be detected above background in these assays, compared to authentic virus neutralization and GFP pseudotyped virus neutralization, where each infectious unit is counted and variance has the same magnitude in both negative and positive samples. Furthermore, SEAP and luminescence detection kits often provide controls and stringent parameters for keeping background noise to minimal levels.

Collectively, these data demonstrate that VSV-ΔG pseudotyped virus neutralization platforms, especially Luci- and SEAP-based platforms, are better at predicting authentic virus neutralization than ELISA regardless of the viral antigen tested. Not only are the Luci- and SEAP-based pseudotype platforms most strongly correlated with authentic virus neutralization, they also have the lowest average difference in log ND_50_ compared to authentic virus neutralization. Previous reports have only compared ELISA titers to pseudotyped virus neutralization (21), ELISA to authentic virus neutralization (23), or only one type of ELISA and one pseudotyped virus platform against authentic virus neutralization (10). Furthermore, integrating the results of multiple serological assays through a principal-component analyses has the potential to better predict authentic virus neutralization than individual assays alone (Fig. 4). Our studies provide one of the most comprehensive comparisons among multiple ELISA antigens, pseudotyped virus neutralization platforms, and authentic virus neutralization.

Of note, several spike- and RBD-positive samples showed very little authentic virus neutralization, despite having moderate to high neutralization on the pseudotyped virus platforms. Furthermore, one sample appeared to show antibody-dependent enhancement (ADE) in the authentic virus neutralization assay (1.8-fold increased PFU), but still showed low but detectable neutralization in all the pseudotyped virus platforms. While there is no definitive role for ADE during human SARS-CoV-2 infection, ADE has been demonstrated *in vitro* with other human coronaviruses (24). Further characterization of this sample and screening for and characterization of similar samples will lead to a better understanding of the risk of ADE during SARS-CoV-2 infection. Recent evidence suggests that several SARS-CoV-2 variants, including B.1.351 and P.1, have decreased neutralization when treated with monoclonal antibodies or polyclonal sera derived from patients infected with early strains of SARS-CoV-2 (25–27). Future studies need to assess how the mutations present in the variants differentially affect ELISA, pseudotyped virus neuralization, and authentic virus neutralization.

In addition to accuracy, the serological assays differ in several key features (Table 4), and the assay of choice may have to be determined by the settings. The requirement for a BSL3 laboratory makes authentic virus assays technically challenging and unfeasible for many clinical and research applications. The high cost of personal protective equipment and facility operations for authentic virus neutralization make it prohibitively expensive for many applications. This can be overcome by pseudotyped viruses. However, creation and validation of the different pseudotyped viruses are not trivial, and readouts may require specialized equipment (e.g., a luminometer for the Luci platform). Additionally, it can be hard to validate the SARS-CoV-2 spike expression levels and ratio of infectious pseudotyped particles, making cross-institution comparison of pseudotyped virus neutralization difficult. Most laboratories have ready access to the equipment needed for performing ELISAs, making the technical requirements for these assays low. ELISAs can also be completed within several hours, while the pseudotyped virus neutralization platforms require 12- to 24-h incubations and authentic virus neutralization requires 48 to 72 h. If all technical requirements have been met and are available, the assays are all relatively inexpensive, except for the Luci platform, which requires expensive reagents for reading the results. If turnaround time is a priority, the RBD and spike ELISAs would provide the fastest results with minor decreases in predicting authentic virus neutralization response. Alternatively, in resource-limited settings like field hospitals, the GFP-based pseudotyped virus neutralization assay requires only a basic fluorescence microscope for readout and is more predictive of authentic virus neutralization than any of the ELISAs. Overall, this study shows that all six serological assays, to various degrees, correlated with authentic virus neutralization, and the optimal serological assay for assessing a protective antibody response is going to be institution and question specific.

## MATERIALS AND METHODS

### RBD/N ELISA.

SARS-CoV-2 RBD protein was diluted to a concentration of 1.5 μg/ml in phosphate-buffered saline (PBS) and added at 50 μl per well to a 96-well ELISA plate. The ELISA plates were sealed and allowed to incubate at 4°C overnight. The next day, the coating solution was removed, and the plates were blocked at room temperature (RT) using 3% milk (200 μl per well) for a minimum of 1 h but not exceeding 4 h. While the plates were being blocked, the samples were prepared by diluting the plasma 1:50 in 1% milk. Following the blocking period, the milk was removed, and the plates were washed 3 times with 0.1% phosphate-buffered saline containing 0.1% Tween 20 (PBS-T) using 200 μl per well. The diluted plasma was added to the blocked plate at 50 μl per well along with 2 positive controls (anti-SARS-CoV-2 RBD antibody at 1:5,000, 1:25,000, 1:125,000, and 1:625,000 dilutions and plasma from a naturally infected donor at a 1:50 dilution) and a known negative, prepandemic plasma sample (1:50). The samples were incubated for 1.5 h at RT and then removed and washed 3 times with 200 μl 0.1% PBS-T. Goat anti-human IgG horseradish peroxidase (HRP)-conjugated secondary antibody was diluted 1:2,500 in 1% milk, and 50 μl was added to each well of the washed plate and incubated at RT for 30 min. Following the incubation period, the secondary antibody was removed, and the plate was washed 3 times with 0.1% PBS-T. *O*-Phenylenediamine dihydrochloride (OPD) substrate was prepared directly before use and added at 50 μl per well for exactly 8 min. The OPD substrate was stopped by adding 50 μl of 3 M HCl, and then the plate was read using a spectrophotometer at 490 nm.

### Spike ELISA.

SARS-CoV-2 spike protein was diluted to a concentration of 2 μg/ml in PBS and added at 50 μl per well to a 96-well ELISA plate. The ELISA plates were sealed and allowed to incubate at 4°C overnight. The next day, the coating solution was removed, and the plates were blocked using 3% milk (200 μl per well) for a minimum of 1 h but not exceeding 4 h. While the plates were being blocked, the samples were prepared by creating a 3-fold serial dilution starting at 1:100 and ending at 1:8,100 (1% milk as diluent). Following the blocking period, the milk was removed, and the plates were washed 3 times with 0.1% PBS-T using 200 μl per well. The diluted plasma was added to the blocked plate at 50 μl per well along with 2 positive controls (anti-SARS-CoV-2 RBD antibody at 1:5,000, 1:25,000, 1:125,000, and 1:625,000 dilutions and plasma from a naturally infected donor at 1:100, 1:300, 1:900, 1:2,700, and 1:8,100 dilutions) and a known negative, prepandemic plasma sample (1:100). The samples were incubated for 1.5 h at RT and then removed and washed 3 times with 200 μl 0.1% PBS-T. Goat anti-human IgG HRP-conjugated secondary antibody was diluted 1:2,500 in 1% milk, and 50 μl was added to each well of the washed plate and incubated at RT for 30 min. Following the incubation period, the secondary antibody was removed, and the plate was washed 3 times with 0.1% PBS-T. OPD substrate was prepared directly before use and added at 50 μl per well for exactly 8 min. The OPD substrate was stopped by adding 50 μl of HCl acid, and then the plate was read using a spectrophotometer at 490 nm. Spike data are presented as either AUC or AUC × 100 in order to plot the data on the same scale as the other ELISAs.

### Tissue culture.

Vero E6 cells stably expressing TMPRSS2 (Vero-TMPRSS2) (XenoTech) were cultured in Eagle’s minimal essential medium (EMEM) supplemented with 10% fetal bovine serum (FBS), 100 U/ml penicillin, 100 μg/ml streptomycin, and 2 mM GlutaMax (Gibco). Medium was supplemented with 1 mg/ml G418 every other passage. All tissue culture was performed in a humidified incubator set to 37°C and 5% CO_2_.

### SARS-CoV-2 neutralizing antibody assay.

Serially diluted plasma samples were mixed with diluted (approximately 6 PFU/cm^2^) SARS-CoV-2 (2019n-CoV/USA_WA1/2020) in EMEM supplemented with 5% FBS, 100 U/ml penicillin, 100 μg/ml streptomycin, and 2 mM GlutaMax. Mixtures were incubated for 1 h in a humidified incubator at 37°C and 5% CO_2_. After 1 h, culture medium was removed from approximately 90% confluent Vero-TMPRSS2 cells grown in 6-well plates and replaced with virus-plasma mixtures. Plates were returned to the incubator for 1 h at 37°C and 5% CO_2_. Plates were rocked manually every 15 min. After incubation, an agarose overlay containing minimal essential medium (MEM) supplemented with 5% FBS, 100 U/ml penicillin, 100 μg/ml streptomycin, 2 mM GlutaMax, 0.075% sodium bicarbonate, 0.01 M 4-(2-hydroxyethyl)-1-piperazineethanesulfonic acid (HEPES), and 1% low-melting-temperature agarose (SeaPlaque; Lonza) was added to each well. Once agarose hardened at RT, plates were returned to the incubator at 37°C and 5% CO_2_. After 48 h, cells were fixed with 10% neutral buffered formalin for 1 h, the agar plugs were removed, and then cells were stained with crystal violet for 5 to 10 min. Upon rinsing with H_2_O, plaques were visualized and counted manually. All samples were run in duplicate, and positive-control plasma (known positive sample), negative-control plasma (known negative sample), and no-plasma controls were run with each batch and used to standardize results.

### VSV-ΔG-GFP–SARS-CoV-2-S neutralizing antibody assay.

Serially diluted plasma samples were mixed with diluted and mixed with spike–VSV-ΔG-GFP pseudotypes in EMEM supplemented with 5% FBS, 100 U/ml penicillin, 100 μg/ml streptomycin, and 2 mM GlutaMax. Mixtures were incubated for 1 h in a humidified incubator at 37°C and 5% CO_2_. After 1 h, culture medium was removed from approximately 90% confluent Vero-TMPRSS2 cells grown in 96-well plates and replaced with virus-plasma mixtures. Plates were returned to the incubator at 37°C and 5% CO_2_. After 24 h, IU were quantified manually using an EVOS fluorescence microscope. All samples were run in duplicate, and positive-control plasma (known positive sample), negative-control plasma (known negative sample), and no plasma controls were run with each batch and used to standardize results.

### Luciferase assay.

Twenty hours prior to assay setup, Vero-TMRSS2 cells were plated in a 96-well plate at 20,000 cells per well in Dulbecco’s minimal essential medium (DMEM) supplemented with 5% FBS and 1 mg/ml G418. For assay setup, plasma samples were initially diluted 1:100 and serially diluted 1:3 in DMEM supplemented with 5% FBS. Diluted samples were mixed 1:1 with spike–VSV-ΔG–luciferase pseudotyped virus diluted to a final 250 IU per well in serum-free DMEM. Mixtures were incubated for 1 h in a humidified incubator at 37°C and 5% CO_2_. After the incubation period, culture medium was removed from Vero-TMPRSS2 cells and virus-plasma mixture was added to the cells in triplicate. Plates were incubated for approximately 18 h in a humidified incubator at 37°C and 5% CO_2_. After the incubation period, Luc-Screen Extended-Glow (Thermo Fisher) buffers were added to the wells according to the manufacturer’s instructions and incubated for a minimum of 10 min at room temperature protected from light. Luminescence was measured with a luminometer using a 1-s integration time.

### SEAP assay.

Twenty hours prior to assay setup, Vero-TMRSS2 cells were plated in a 96-well plate at 20,000 cells per well in DMEM supplemented with 5% FBS and 1 mg/ml G418. For assay setup, plasma samples were initially diluted 1:100 and serially diluted 1:3 in DMEM supplemented with 5% FBS. Diluted samples were mixed 1:1 with purified spike–VSV-ΔG–SEAP pseudotyped virus diluted to final 250 IU per well in serum free DMEM. Mixtures were incubated for 1 h in a humidified incubator at 37°C and 5% CO_2_. After the incubation period, culture medium was removed from Vero-TMPRSS2 cells and virus-plasma mixture was added to the cells in triplicate. Plates were incubated for approximately 28 h in a humidified incubator at 37°C and 5% CO_2_. After the incubation period, Quanti-Blue (InvivoGen) solution was combined with 20 μl supernatant according to the manufacturer’s instructions and incubated for a minimum of 15 min at 37°C protected from light. Optical density was measured at 620 to 655 nm.

### SARS-CoV-2–VSV pseudotype production.

VSV-ΔG pseudotypes displaying the full-length SARS-CoV-2 spike (Wuhan-Hu-1 strain) were generated essentially as described previously (28) with the following modifications. Baby hamster kidney (BHK-21) cells in 10-cm dishes were transfected using Lipofectamine 2000 according to the manufacturer’s instructions with 24 μg of a plasmid encoding a codon-optimized cDNA for the SARS-CoV-2 spike (20), which was generously provided by Florian Krammer. Approximately 20 to 24 h later, the transfected cells were infected at a multiplicity of 5 with VSV-G pseudotyped ΔG-GFP, luciferase, or SEAP. Virus was adsorbed for 1 h, the inoculum was removed, cells were rinsed once with serum-free DMEM, and then 4 ml of hybridoma supernatant containing the I1 monoclonal antibody (29) was added for 30 min to neutralize residual VSV-ΔG pseudotyped virus from the inoculum and then replaced with DMEM containing 20% fetal bovine serum. The supernatant containing the spike-ΔG pseudotypes was collected 22 to 24 h later, cell debris was removed by centrifugation at 450 × *g* for 10 min. For the ΔG-GFP and luciferase pseudotypes, the supernatant was aliquoted and stored at −80°C. For the ΔG-SEAP pseudotypes, the supernatant was transferred to a Beckman SW41 tube, underlayered with sterile 20% sucrose in PBS, and virus was pelleted at 35,000 rpm for 45 min in a SW41 swinging bucket rotor. Pelleting virus was required to separate it from SEAP released from the infected cells. The pellets were resuspended in DMEM containing 20% FBS and stored at −80°C.

### Statistics.

Area under the curve (AUC) and 50% neutralization dilution (ND_50_) analyses were performed in GraphPad Prism (version 9.0.0): nonlinear regression (dose response, agonist versus normalized response). Fifty percent effective concentration (EC_50_) values were inverted to generate ND_50_ values. Pearson’s *r* values for comparing assays by percentage of maximum AUC were calculating using simple linear regression analysis in GraphPad Prism. AUC and ND_50_ values for the different assays were compared by mixed-effects model with the Geisser-Greenhouse correction and Tukey multiple-comparison posttest and *P* value adjustment in GraphPad Prism (version 9.0.0). Kruskal-Wallis tests with Dunn's multiple-comparison tests. were performed to compare neutralizing antibody responses between highly positive ELISA samples, low-positive ELISA samples, and negative samples. Principal-component analysis (PCA) was performed in GraphPad Prism (version 9.0.0) with principal components selected based on parallel analysis. A 95% percentile level was used, and 1,000 simulations were performed for the PCA. The Bland-Altman analyses were performed in GraphPad Prism (version 9.0.0).
